# Acknowledgement to Reviewers of *IJERPH* in 2017

**DOI:** 10.3390/ijerph15010118

**Published:** 2018-01-11

**Authors:** IJERPH Editorial Office

**Affiliations:** MDPI AG, St. Alban-Anlage 66, 4052 Basel, Switzerland

Peer review is an essential part in the publication process, ensuring that *IJERPH* maintains high quality standards for its published papers. In 2017, a total of 1594 papers were published in the journal. Thanks to the cooperation of our reviewers, the median time to first decision was 22 days and the median time to publication was 53 days. The editors would like to express their sincere gratitude to the following reviewers for their time and dedication in 2017:

Aadland, EivindLeonhard, ChristophAasvang, Gunn MaritLépy, ÉliseAbballe, AnnalisaLercher, PeterAbbott, LouiseLerchl, AlexanderAbiGhannam, NiveenLê-Scherban, FéliceAbimanyi-Ochom, JulieLeung, KatherineAblewhite, JoanneLeung, JanniAboud, FrancesLevallois, PatrickAbraham, John P.Levi-Belz, YossiAbraham, K. J.Levizou, EfiAbrams, DavidLewinson, TerriAbrams, StephenLewis, KathleenAdam, VojtechLi, ZijianÁdám, BalázsLi, WeidongAdamus-Białek, WiolettaLi, DongmeiAddison, CliftonLi, SiweiAddo, O. YawLi, ChenyuAdetona, OlorunfemiLi, DapengAdini, BruriaLi, XuemeiAdivar, BurcuLi, SenAdler-Nissen, JensLi, FuzhongAdoloratto, GiovanniLi, Xiangyu (Dale)Afshari, RezaLi, YonghuaAggarwal, AnjuLi, JianghongAggazzotti, GabriellaLi, YongjianAgho, KingsleyLi, XuebingAgnisola, ClaudioLi, XiujunAgodi, AntonellaLi, BaoguangAgrawal, PriyankaLi, ShandyAguilar, CarmeLi, YingAguinaga-Ontoso, InésLi, WeijuanAhern, TraceyLi, RuiAhmad, Hafiz AnwarLi, ZoeAhmad, AzeemLian, TinaAhmed, FaruqueLian, Ie-BinAhmed, ZafarLiang, JuneAi, ZhengtaoLiang, YatingAida, JunLiao, HaifengAide, Michael T.Liao, YungAigner, RafaelLichand, GuilhermeAikins, DeaneLicitra, GaetanoAitken, DominicLiddle, BrantleyAitor, Nogalez-GonzalezLieberman, Lauren JoyAittasalo, MinnaLiew, ZeyanAkbari, Mohammad RezaLiguori, GiorgioAkin, Becci A.Liller, KarenAkpinar, AbdullahLim, HyeyeunAlarco, JoseLim, Keah YingAl-Banna, Nadia A. M.Lim, MeganAlbert, Steven M.Lin, WeiAlberti, LucaLin, Thung-hongAlbini, AngeloLin, Jin-DingAlbornoz, Emilio Morales Carrillo DeLin, YeAlbrecht, HuguetteLin, ShengdaAlcock, LisaLin, Ken-YuAlda, NataleLin, Pao-HwaAldieri, LuigiLin, Chiu-YueAldrich, Rosalie S.Lin, Yi-ChinAldstadt, JaredLin, JonqlanAleluia Da Silva Reis, LaraLin, ZhoumengAlemany, SilviaLin, XiongAlemi, QaisLin, Pao-HwaAletras, VassilisLin, Ming-TaiAletta, FrancescoLin, YanAlexander, DavidLindahl, JohannaAlexeeff, StaceyLindert, JuttaAlfonso, Jose HernanLindholm, DanAlfonso-Sierra, EduardoLindong, IanAlhabash, Saleem E.Lindquist, MarkAli, OmarLindsay, RyanAliev, FazilLink, DeniseAllegaert, KarelLiou, JamesAllem, Jon-PatrickLipnicki, DarrenAllen, DavidLisa, AntonellaAllen, VivietteLitescu, Simona CarmenAlley, StephanieLitman, ToddAllik, MirjamLittle, JulianAlmberg, Kirsten StaggsLiu, Pei-YangAlmeida, Maria Adelaide AraújoLiu, Jia CocoAlmeida Palha, JoanaLiu, Li-FanAlsharairi, NaserLiu, Kevin Fong-ReyAlshbool, Fatima Z.Liu, XinAlves, ElisabeteLiu, Yu WeiAlves, LuisLiu, ChengAlves-Pereira, MarianaLiu, Hui-MingAmagai, TeruyoshiLiu, JieAmarasena, NajithLiu, ChiangAmaro, AnaLiu, BarbaraAmbrey, ChristopherLiu, Chiung-JuAmbrosino, ElenaLiu, YongAmer, SherifLiu, GeorgeAmini, RezaLiu, Chien-TsaiAmirkhanian, Yuri A.Liu, AijunAmmerman, AliceLiu, ZijuanAmor, Antonio J.Liu, GuoxiangAmrith, MeghaLiu, ChunAnaby, DanaLiu, BinAnafi, PatriciaLiu, Ko-FeiAnagnost, Susan E.Liu, ChengfangAnanga, ErickLiu, JinglingAnastos, KathrynLiu, Hai-YingAndersen, Helle R.Liu, YuweiAnderson, Britta L.Liu, ZhaorongAndersson, Els-MarieLiu, PeideAndjelkovic, MirjanaLiu, GangAndo, TakaoLiverani, SilviaAndrade, VandaLivingston, MarkAndreassi, Maria GraziaLivingston, MichaelAndreeva, ElenaLlorens, JordiAndreoli, RobertaLlorens, EugenioAndreopoulou, ZacharoulaLo, Huang-MuAndrew, MelissaLoaiciga, Hugo A.Andrews, ArthurLochbaum, MarcAnenberg, SusanLockie, RobertAngelidis, ApostolosLoerbroks, AdrianAngelone, TommasoLogsdon, SallyAngermann, JeffLöhmus, MareAnifandis, GeorgeLokkerbol, JoranAnna, OdoneLomonaco, GuglielmoAnnunziata, AzzurraLonardo, AmedeoAnsa, BenjaminLong, Thomas B.Antar, AlliLonga, Claudia Maria OliveiraAnthony, TracyLopez, NanetteAntipova, AngelaLopez Jornet, PiaAnton, FernandLópez López, DanielAntonanzas, FernandoLopez-Aparicio, SusanaAntoñanzas, FernandoLópez-Fernández, MacarenaAntonarakis, Gregory S.López-Vizcaíno, RubénAntosiewicz, Danuta MariaLoppi, StefanoAnttila, AhtiLorber, MatejaAppell, MichaelLorber, SophieAraki, AtsukoLoss, JulikaArakida, MikakoLoton, DanAranzabal, AsierLou, SongArchambault, StevenLouis, JosephArchibald, DaryllLoureiro, AnaArcila Calderón, CarlosLoury, SharonArcury, Thomas A.Love, TanzyArenas Moreno, AliciaLovreglio, PieroArias, Ana VictoriaLow, JacquelineAriese, FreekLöytönen, MarkkuArmstrong, PatLozano-Sufrategui, LorenaArola, AnttiLu, GuiningArora, AmitLu, YuArrandale, Victoria H.Lu, YiArrebola, Juan P.Lu, BinbinArreola, SonyaLuan, HuiArthur, FrankLuben, TomArturo, GoldarazenaLucas, Paul L.Asimakopoulos, AlexandrosLue, FangAslan, AsliLugg-Widger, FionaAspray, ThomasLugo, AlessandraAssari, ShervinLuick, Bret R.Assimakopoulos, Vasiliki D.Luís, AnaÅström, ChristoferLukaszuk, JudithAtkinson, SamLuna, Gian M.Atti, Anna RitaLund, SandyAuger, NathalieLundälv, JörgenAugsburger, MareikeLundeen, ElizabethAuyeung, Tung WaiLuo, PingpingAvellán, TamaraLuo, Xiao-SanAverill, MichelleLuo, YanAvraam, DemetrisLusa, MerjaAycock, DawnLusk, AnneAyonrinde, OyekoyaLyall, KristenAyo-yusuf, Olalekan A.Ma, YiyiAysa-Lastra, MariaMa, XiaoguangAzeez, AdeboyeMa, Zheng FeeiAznar, SusanaMa, PengBabatunde, OpeyemiMa, LiBabatunde, EmmanuelMa, GuanshengBabu, Giridhara R.Mabuchi, KiyohikoBacciu, ValentinaMacArthur, GeorginaBaciu, CălinMacCarthy, MichaelBacker, Charlotte DeMach, PavelBackes, ToddMachado, CarolinaBadgley, BrianMachell, GeorgiaBađun, MarijanaMacher, Janet M.Bae, HaroldMackinnon, GillianBahnfleth, William P.MacLeod, Catherine A.Bahwere, PalukuMacMillan, FreyaBaigorri, RobertoMacNell, LillianBailey, KylieMacy, Jonathan T.Bailey, CatherineMadonia, AliceBaird, NicholasMadsen, TrineBalachova, Tatiana N.Maeda, TakafumiBalastegui, A.Magee, MadelineBalawejder, MaciejMagnani, CorradoBalcazar, Hector G.Magnavita, NicolaBalcom, PrentissMaguire-Jack, KatieBaldacchino, FredericMah, CatherineBaldasano, Jose M.Maharaj, SaviBallesteros, MickMaher, Jaclyn P.Balocco, CarlaMaher, ChristopherBalouch, SaraMaher, WilliamBalter, VincentMahmud, Khan M.Balwicki, ŁukaszMai, LuBan, MasahitoMaicaneanu, Sanda AndradaBana, JinanMaier, BirgaBanaitis, AudriusMain, GillBanerjee, AreenMainka, AnnaBanik, Gouri RaniMaisha, BuumaBaragetti, AndreaMajeed, BanBarbara, PancinoMajewska, Maria DorotaBarcelos, AnabelaMajumdar, IndrajitBarclay, LindaMaksymiuk, GabrielaBarker, Bridget M.Malandrino, MeryBarman, ApurbaMalara, AlbaBarnes-Josiah, DeboraMalecki, KristenBaron, KellyMaleknia, Simin D.Bar-Or, DavidMalesios, ChrisovalantisBarranco Ruiz, YairaMalhotra, PraveenBarrington, DaniMali, MatildaBarron, SusanMalissiova, EleniBarros, NillaMallow, OleBarta, CsengeleMalone, Susan K.Bartlett, DelwynMambretti, StefanoBartlo, PamelaMamen, AsgeirBartoli, FrancescoMamudu, HadiiBartolucci, Giovanni BattistaMamudu, Hadii M.Barton-Forbes, MichelleManandhar, RejinaBartzis, JohnMancini, FrancescoBasile, GianpaoloManderscheid, Ronald W.Baska, TiborManikas, Andrew S.Basta, Nicholas ThomasManjunath, ManuboluBasu, PratyushaMann, Samuel JosephBatey, ScottMannucci, Pier MannuccioBathula, ChandraMansourian, AliBatista Henriques, IzabelaManzo, CiroBattaglin, William A.Mao, LiangBaudry, GinoMarano, NinaBaugerud, Gunn AstridMarchant, DavidBaumgardner, DarrelMarchi, MichelaBaumgardner, Dennis J.Marczylo, EmmaBaumgardt, JohannaMargaritis, LukasBaxter, LisaMárialigeti, KárolyBayat, SaharMari-Dell’Olmo, MArcBayer, Ilker S.Marin, DanielaBayer, Frank O.Marino, JosephBazzani, ClaudiaMarkevych, IanaBcheraoui, Charbel ElMarks, RayBeaty, TerriMarreilha Dos Santos, Ana PaulaBeaumont, RobinMarrs, CarlBeauvais, Wendy A.Marston, LouiseBeckman, LindaMartin, MichelleBednarczyk, Robert A.Martin, Richard H.Beery, TomMartin, JeffBeil, KurtMartinez, Antonio J. TorijaBeintner, InaMartínez, Ramón PeñaBeizaee, ArashMartinez-Camblor, PabloBelan, IngridMartinez-Colon, MichaelBelay, BrookMartínez-Guitarte, José-luisBELIN De Chantemèle, Eric J.Martinez-Molina, AntonioBelknap, RobertMartins, Maria Luisa LouroBell, SimonMartins, MariaBell-Anderon, KimMarzano, LisaBellinger, DavidMascherini, GabrieleBellotti, ElisaMaslow, Joel N.Bełtowski, JerzyMason, Lisa ReyesBend, JackMassaccesi, LuisaBenitez Arciniega, AlejandraMassas, IoannisBenjamin-Garner, RubyMasten, SusanBennett, JamesMasterson, ErinBennett, JulieMasuda, CamlynBenning, JenniferMatanle, PeterBentley, GillianMateo-Garcia, MonicaBento, Douglas MayerMatheson, AnnaBerardino, SantinoMathew, SupriyaBerchialla, PaolaMathews, Anne E.Bergamaschi, EnricoMathur, ManuBergen, DorisMatsubara, KeiichiBerger, MatthiasMatsuda, KazuhikoBerger, Ann M.Matsui, KenjiBergeron, KimMatsumoto, Ken-ichiroBerglund, ErikMatud, María PilarBergouignan, AudreyMatz, Carlyn J.Berkhof, JohannesMauny, FredericBerkowsky, RonaldMaupomé, GerardoBerland, AdamMaurer-Fazio, MargaretBerli, CorinaMavaddat, NahalBerliner, ElizabethMavromichalaki, HelenBerman, TamarMawson, Anthony R.Bernabò, NicolaMaxim, L. DanielBernardi, GiulioMayer, AdamBernardini, GiuliaMaynard, Olivia M.Bernstein, Eve R.Maynard, Leah MicheleBerrington, JanetMayner, LidiaBerry, MeredithMazumdar, SumitBerry, PeterMazur, JoannaBerry, William R.Mbulo, LazarousBerry, NarelleMcBride, Devin W.Berry, TanyaMcCabe, BrianBertocci, MicheleMcCaffrey, NikkiBertoni, GiuseppeMcCann, KatharineBertram, ChristineMcCarthy, ValerieBertrand, JaneMcCormack, LaceyBesenyi, GinaMcCormick, SabrinaBetz, MarianMcCoy, Sarah J. BreeseBeute, FemkeMccoy, StephanieBeutell, Nicholas J.McCreesh, NickyBezner, JanetMcCullugh, CathyBhalla, NikhilMcDermott, SuzanneBhatta, RheaMcdonald, TedBhaumik, AnusarkaMcEachan, RosieBhowmik, ArnabMcElroy, JaneBhuiyan, AzadMcFadden, JonathanBiagi, FedericoMcGovern, Patricia M.Bian, JunsongMcGregor, CarolynBianchi, Anna MariaMcGuire, SarahBianchi, FabrizioMcKay, CarlyBias, ThomasMcKeganey, NeilBibbo, JessicaMcKendry, CorinaBickham, David S.McKenzie, EricaBidasee, KeshoreMcKenzie, LisaBiddle, StuartMcKenzie, PaulBidmead, ElaineMcKinley, JenniferBierens, JoostMcKinley, MichaelBiernasiuk, AnnaMcKinstry, CarolBihuniak, Jessica D.McLaughlin, John E.Bilgin, AysenurMcLeod, Geraldine F. H.Billiard, MichelMcLeroy, KennethBin, Yu SunMcLuskey, JohnBind, Marie-AbèleMcMorris, TerryBinfet, John TylerMcQuade, Kevin J.Bingham, DanielMcQuaid, Christopher FinnBinns, JohnMeacham, Susan L.Binyamin, JacquelineMears, Stephen A.Birch, Stephen (Steve)Meddings, DavidBirch, BrianMedina-Vera, MyriamBisbal, CatherineMeerlo, PeterBischoff, KarynMehmood, AmberBisharat, NaielMehta, TapanBiziuk, MarekMei, ChuanshengBlack, LeonMeijer, ElineBlack, CarlaMelander-Wikman, AnitaBlackall, PatrickMelendez, RitaBlackmore, CarinaMelo Zurita, Maria De LourdesBlackmore, ChrisMeloni, ItaloBláha, LadislavMendelsohn, ColinBlair, PeterMendes, Ana Sofia EstevãoBlanchette, MelanieMendes, Maria PaulaBlaschke, PaulMéndez-Lázaro, Pablo A.Blažíčková, StanislavaMercer, JennyBleiweiss, MaxMercille, JulienBlock, DanielMerget, RolfBlock, Martin E.Merino, GraciaBlukacz-Richards, E. AgnesMerklinger-Gruchala, AnnaBlum, Igor RMerrick, TeresaBlumer-Schuettea, Sara E.Merry, MelissaBlüml, VictorMethner, UlrichBoan, DavidMeyer, StephenBoard, MaryMeyer-Rochow, Victor BennoBoardman, JedMézes, MiklósBoaz, MonaMeziti, AlexandraBöckmann, MelanieMguni, PatienceBodin, JohannaMi, ZhifuBodin, TheoMian, CaterinaBogdanovica, IlzeMichaelidou, Alexandra-MariaBogunovic, IgorMichailidis, PanagiotisBojke, ChrisMichalke, BernhardBolton, JamesMichelozzi, PaolaBondevik, Gunnar TschudiMichikawa, T.Bonetta, SilviaMicu, AdrianBonetta, SaraMiddleton, NickBongaerts, BrendaMidgley, AdamBoniface, SadieMielgo Ayuso, JuanBoomer, Jonathan S.Mielke, HowardBoomhower, JudsonMier, NeldaBoquien, Clair-yvesMignon, SylviaBorde, ThedaMikkelsen, Bent EgbergBorena, WegeneMikkers, M. C.Borisenkov, Mikhail F.Mildorf, TomasBornman, LizaMilic, NatasaBorras, Josep M.Milici, Frances FlemingBorrego, CarlosMilla, SaajanahoBorrmann, ThomasMiller, Judith S.Borschmann, RohanMiller, CharlesBortkiewicz, AlicjaMiller, Emma R.Borzabadi-Farahani, AliMiller, ShellyBos, PeterMin, LeileiBos, ElskeMingo, AntonioBosarge, Patrick L.Mínguez-Alarcón, LidiaBoshuizen, HendriekMinicozzi, PamelaBosma, HansMir, DebbyBossew, PeterMiraglia, MariellaBossi, GiuliaMirauda, DomenicaBosso, LucianoMirlohi, SusanBotella, Miguel C.Mirman, Jessica HBothe, DeniseMiron, Isidro JuanBouaïcha, NoureddineMisciagna, GiovanniBouamra, OmarMita, Damiano GustavoBourgeois, Denis M.Mitchell, Joshua J.Bourliva, AnnaMitchell, LanaBowatte, GayanMitchell, DanielBowen, Deborah J.Mitchell, RodBower, Kelly M.Mitkowski, PiotrBoyas, Javier F.Mitrou, FrancisBrackbill, Robert M.Miwa, TomioBrahmbhatt, Tejal S.Miyamoto, KuniakiBraks, MarietaMiyoshi, Shin-ichiBranco, Manuel CasteloMizuno, KohBrands, EdwinMizuno, TooruBrantsaeter, Anne LiseMo, LingfeiBratkic, ArneMo, JinhanBrattich, ErikaMohatt, NathanielBråtveit, MagneMohebbi, MohammadrezaBrauner-Otto, Sarah R.Möhner, MatthiasBrčić Karačonji, IrenaMoise, Imelda K.Breitenfeld Granadeiro, LuizaMokkenstorm, JanBrekke, IdunnMole, RichardBremberg, SvenMoliner-Urdiales, DiegoBreton, MylaineMolino, MonicaBrewer, George J.Mollerup, SteenBridle-Fitzpatrick, SusanMomtaz, SalimBrien, James D.Moncada, StefanoBrimblecombe, PeterMondal, DebapriyaBrinthaupt, Thomas M.Mónica, Perez Bazán LauraBritch, Seth CarrollMonroe, KathyBritner, Preston A.Monroy, FernandoBrittain, KellyMonsur, MuntazarBroadbent, Jonathan M.Montes, AgustínBrokamp, ColeMontgomery, JoLynn P.Bronzaft, ArlineMontllor, JoanBrookfield, KatherineMontuori, PaoloBrooks, SteveMonyeki, KotsediBroom, IainMoon, Christopher J.Broome, KieranMooney, SandraBrough, LouiseMoore, Simon C.Brown, PhilMoore, PaulBrown, Scott C.Moore, GeromyBrown, DavidMoore, KarenBrown, PatrickMoosmann, BerndBrown, David M.Mora, RodrigoBrown, Richard J. P.Morales Suárez-Varela, María M.Brown, JulieMorawa, EvaBrownstein, NaomiMoreno, TeresaBrunt, DavidMorera, MariaBruschi, FabrizioMorgan, MarshaBrusselaers, NeleMorin, CoryBrymer, EricMorioka, TomoakiBrzoska, PatrickMorita, ManabuBubici, GiovanniMoriya, ShingoBuchanich, Jeanine M.Morphett, KylieBudde, HenningMorrell, Holly E. R.Buitrago, María JoséMorris, CodyBujnowska-Fedak, Maria MagdalenaMorris, MelanieBunaciu, Rodica P.Morris, Robert D.Bungum, TimothyMorrissey, JoannaBurden, SandyMorrone, MicheleBurgess, John A.Morrow, JenniferBurgoine, ThomasMoskalewicz, JacekBurinskienė, MarijaMosnier, AnneBurkart, KatrinMoss, AntonyBurke, Rita V.Mouchtouri, Varvara A.Burke, Ronald J.Mougiakakou, StavroulaBurkhart, Craig G.Moustris, KonstantinosBurkowska-But, AleksandraMoynier, FrédéricBurlage, RobertMozumder, MohammadBurnett, EmmaMpofu, EliasBurris, ScottMrhálek, TomášBurrows, StephanieMücke, Hans-GuidoBurszta-Adamiak, EwaMudde, AartBurzynska, MonikaMudge, SuzieBus, James S.Mueller, Megan P.Busby, ChrisMuggeo, Vito M. R.Buscarinu, Maria ChiaraMui, Kwok-waiBush, AndrewMulia, NinaBusija, LucyMüller, Jochen A.Busjahn, AndreasMüller, K. W.Butler, Matthew P.Müller, JohannesButler, JohnMüller, RuthButner, JonathanMulligan, HildaButtafuoco, GabrieleMulligan, KateButtiglieri, GianluigiMullin, James M.Buvik, KristinMulvaney, Caroline A.Byeon, Sang-HoonMundorf, ChrisByon, Ha DoMundorf, ChristopherByrne, MiriamMundt, SabineBywaters, PaulMunir, MariyaCabello, MariaMunoz, MacarenaCabral-Pinto, Marina M. S.Murakami, KeikoCabrerizo, Francisco JavierMuramatsu, NaokoCaccamo, DanielaMurante, Anna MariaCacciottolo, MafaldaMurbach, ManuelCaesens, GaëtaneMurff, SharonCaiado, CamilaMurphy, DominicCaiani, EnricoMurphy, Georgina A. V.Callahan, Damien L.Murray, MeganCallison, KevinMurtaugh, MaureenCalogiuri, GiovannaMusaad, SalmaCalvo, ArleneMushtaq, NasirCalvo, AngelaMusk, BillCalvo, ConcepciónMusliner, KatherineCamacho, LuisaMusmarra, DinoCamacho-Miñano, Maria JoséMutoh, MichihiroCamenisch, Todd D.Mutti, LucianoCamerino, DonatellaMydlarz, CharlieCamiciottoli, GiannaMyers, CandiceCamille, PerchouxMyrberg, GunnarCampa, A. M. Sa′nchez De LaNa, HuiminCampana, RaffaellaNadais, HelenaCampbell, Frances A.Nagayoshi, MakoCampbell, MarilynNaha, Pratap C.Campbell, CoreyNahar, VinitaCampbell, Katarzyna AnnaNahar, Vinayak K.Campbell Jenkins, Brenda W.Nakamura, MasashiCampbell-Arvai, VictoriaNangia-Makker, PratimaCampo, LauraNanjappa, ManjunathaCampobasso, Carlo P.Nanos, NikosCamporeale, RosaliaNante, NicolaCandice, Young-RojanschiNapiórkowska-Krzebietke, AgnieszkaCangussu Da Silva, Lucia ReginaNarasimhan, SukanyaCantrell, JenniferNardell, EdwardCao, Yong-ChangNash, PoppyCao, HaiqunNash, SarahCao, YongxiaoNassan, FeibyCao, Gui-YingNavacchia, Maria LuisaCao, GuangyuNavarrete-Muñoz, Eva MariaCapolongo, StefanoNavarro, JoseCapolupo, AlessandraNavarro Olivas, RaúlCappella, Joseph N.Nawrocka, AgnieszkaCapra, Gian FrancoNaylor, LindsayCaravanos, JackNaz, SabrinaCardemil, EstebanNazeer, MajidCarey, ReneeNazir, SalmanCarignan, CourtneyNduka, JohnCariñanos, PalomaNduna, MzikaziCarling, Gregory T.Negrón, RosalynCarlisle, Gretchen K.Nelson, Lindsay D.Carlo, Piero DiNemeth, LynneCarlson, Jordan A.Nenna, RaffaellaCarlsson, MariaNeslund-Dudas, ChristineCarlton, JillNeto, Maria AugustaCarmine, GuarinoNeumann, TimCaron, RosemaryNewall, Emma RochelleCaron, Jeffrey G.Newbold, K. BruceCarr, SamNewby, RuthCarr, SherileneNewman, John F.Carré, VincentNewman, Alan P.Carrero, Jose AntonioNewton, JohnCarrillo, GennyNeymotin, FlorenceCarroll, Suzanne J.Ng, Chris Fook ShengCarson, Tiffany L.Ng, Lisa C.Carta, FabrizioNg, Isabella F. S.Cartalis, ConstantinosNg, KwokCarter, WayneNguyen, Ruby H. N.Carter, EricNi, DebingCarugno, MicheleNicholas, JenniferCaruso, BethanyNicholls, KeithCaruso, Joseph A.Nichols, Michelle G.Carvalho, Anna C. C.Nie, PengCarvalho, António PauloNieboer, EvertCasanovas, CarmenNiebylski, MarkCasas, IreneNiedzielski, PrzemysławCaserta, DonatellaNiehoff, Jens-UweCasey, PatriciaNielsen, Samara JoyCaspi, CaitlinNiemann, LarsCassarino, MaricaNieminen, PenttiCassidy, SophieNieves, KourtneyCassidy, LauraNijmeijer, ArianCastellanos, PaolaNikitas, AlexandrosCastellazzi, Anna MariaNikolaidou, MariaCastelli, Darla M.Nobmann, Elizabeth D.Casteren, Jan VanNolan, Laura B.Castro, Shamyr SulyvanNoli, FotiniCattani, GiorgioNomura, KyokoCavallini, GiorgioNoonan, RobertCaxaj, SusanaNoordam, RaymondCayón De Las Cuevas, JoaquínNori-Sarma, AmrutasriCazzato, EugenioNorman, TrudyCerchione, RobertoNormanno, GiovanniCerniglia, LucaNorra, ChristineCervellin, GianfrancoNorval, MaryCésar, CarinaNoszticzius, ZoltánCetin, MehmetNotley, CaitlinCha, Christine B.Notredame, Charles-EdouardChadborn, NeilNovack, TessioChadwick, PhilipNovellino, AlessandroChafe, Zoe AnnaNowak, GlenChai, SangmiNowak, Michal S.Chai, Tian FengNowalk, Mary PatriciaChai, XingyunNowicka, PaulinaChakraborty, JayajitNowicka, GrażynaChalbot, Marie-Cecile G.Nowotny, NorbertChamine, IrinaNshimyimana, Jean PierreChan, MichaelNübling, RüdigerChan, Y. K.Nüesch-Inderbinen, MagdalenaChan, Yu-YiNugraha, AdiChan, Catherine B.Núñez-Delgado, AvelinoChan, Alan H. S.Nwanodi, OromaChandran, ArunaNyangweso, MaryChandrasiri, UpekshaNyholm, MariaChang, Chien-ChungNyrud, Anders Q.Chang, Po-JuO´Higgins Norman, JamesChang, Chih-ChingO’dwyer, LiselChang, Chen-KangO’Hare, LiamChang, Hsien-YenOberbeckmann, SonjaChang, KaowenOblad, TimothyChang, Kyu-SikObolewski, KrystianChang, Geng-RueiO’Brien, Karel K.Chang, Hsien-YenOchiai, TsuyoshiChang, Wen-DienO’Connor, RichardChang, Ta-YuanOctaria, RanyChang, Shu-ManOdland, Jon ØyvindChao, How-RanO’Dwyer, JeanCharisiadis, PantelisOesterle, SabrinaCharles, Luenda E.Ogeil, RowanChatterjee, AvikOgórek, RafałChaves, FabioO’Hare, AnthonyChe, HuizhengOhnishi, MasatoshiChe, WeitaoOhtsuru, AkiraChelbi, Sonia T.O’Keefe, DeniseChen, Jo-HuiOkoro, ChinyereChen, JiangangOkuda, MasayukiChen, ChunOkulicz-Kozaryn, AdamChen, Yi-MinOlafsdottir, GunnthoraChen, MaosiOlde Rikkert, Marcel G. M.Chen, ShumeiOliveira, TeresaChen, LvOliveira, Paula Alexandra Martins DeChen, Yi-ChunOliveira, Cláudia SirleneChen, Mei-LanOlivier, JillChen, HuaiHOlsen, JonathanChen, WenhaoOlu, OlushayoChen, BoOluwole, OluwafemiChen, ZhuoOmoruyi, FelixChen, Jui-ShengO'neill, EoinChen, YaoningOnicescu, GeorgianaChen, Xiang (Peter)Opaas, MarianneChen, Kow-TongOrbea, AmaiaChen, Joyce Chiao-NanOrecchio, SantinoChen, XiangOrmandy, DavidChen, WeihongOrnelas, IndiaChen, KaiOrnelas, YoshiraChen, WeiOrosa, JoséChen, Wei-HsiangOrpana, HeatherChen, Jeff Yi-fuOrtega, EnriqueChen, Tuo-YuOrtega, JohnChen, ChehongOrtiz-Silla, RoqueCheng, TerenceOrton, SophieCheng, Kai-WenOrton, ElizabethCheng, AnOsborne, NicholasCheng, EvanOsin, Evgeny N.Cheong, Hae-kwanOsman, Alfatih A. A.Cherniack, Evan PaulOsteen, PhilipCheung, Yin TingOstrowski, Lawrence E.Chhatwal, JugeshOta, AtsuhikoChi, Kai HsienOtero, MartaChiabai, AlineO’Toole, TimChiavegatto Filho, AlexandreOtsuki, TakemiChiefari, EusebioOtte, DietmarChien, Chun-RuOu, JudyChien, Yi-WenOu, YichangChih, Ming-YuanOu, ChunquanChilds, EmmaOudin, AnnaChileshe, NicholasOuyang, JianmingChillón, PalmaOvalle-Perandones, Maria AntoniaChin, Cheng SiongOviedo Ocaña, Edgar RicardoChiou, Tzeon-JyeOwusu, AndrewChiou, Yuan-YowOzaki, NoriatsuChipps, JenniferOzawa, MioChisholm, JuneOzbay, GulnihalChitchumroonchokchai, ChureepornOzoe, YoshihisaChiu, Yu-LingPääkkönen, RaunoChiu, Yung-hoPaap, Kenneth R.Chiu, Chung-YiPaavilainen, EijaChiva-Blanch, GemmaPacek, LaurenChmielowski, KrzysztofPachego-Torgal, FernandoChmurzyńska, AgataPadula, AmyCho, ClarePaganotti, Giacomo MariaCho, Do YeonPage, KristenCho, MihyePai, Chi-WeiChoate, Peter W.Palagyi, AnnaChobot, VladimirPalazzolo, Dominic L.Choi, KelvinPalmer, Jack A.Choi, EdmondPalmer, Raymond F.Choi, Young MinPalmisano, GiovanniChoi, Hyun-DeokPalsamy, PeriyasamyChoi, Jung-SeokPaludi, MicheleChong, MarcPammolli, AndreaChow, Bik ChuPampel, FredChrastný, VladislavPan, MeixiaChrisinger, BenjaminPan, XiChristiansen, HannaPan, ZhujunChristoph, RupprechtPan, JiayanChristophe, WaterlotPan, Ming-JuChrzanowski, ŁukaszPanagiotidi, MariaChu, Chun-HungPanagiotopoulos, PanagiotisChu, ChunliPang, Chan SiewChua, Tze-ErnPang, Myung-GeolChuang, Ting-WuPani, Shantanu KumarChuang, Hung-YiPanicucci Zíková, AlenaChuang, Shu-ChunPankratz, CurtisChuang, Kai-JenPant, PallaviChuang, Li-MinPanza, FrancescoChuang, Wen-ChingPapadakis, RaffaelloChui, HelenaPapadimitriou Olivgeris, MatthaiosChung, Hsin-FangPapadimitriuou, KonstantinosCiccacci, CinziaPapadopoulos, NestorasCiccone, Marco MatteoPapadopoulou, ChrissanthyCiclitira, PaulPapapostolou, VasileiosCidro, JaimePapazafiropoulou, Athanasia K.Cieplak, MaciejPape, KristineCiesielczuk, TomaszPape, Hans-ChristophCihacek, LarryPappas, Richard StevenCilluffo, GiovannaPaquet, CatherineCipollina, MariaParajuli, Rajendra PrasadCissé, GuéladioParajuli, RajendraClaassen, LiesbethParazzini, MartaClair, AmyPardío-Sedas, Violeta T.Clark, GaryParent, André AnneClark, Thomas A.Parise, Carol A.Claßen, ThomasParisi, MimmoClayton, NicolaParisi, AlfioCleland, VerityPark, Il-KwonClements-Croome, DerekPark, SubinClimie, RachelPark, BoyoungClivot, HuguesPark, SojungClose, Dan M.Park, Jong-IkCoelho, Luis Miguel SerraPark, Sook-HyunCoetzer, TheresaPark, Jae BumCohen, AaronPark, VanCohen, Harold C.Parke, AdrianCole, RoyParker, KimberlyColeman, MaryParker, StaceyColeman, William B.Parlesak, AlexandrColicino, ElenaParr, EvelynColleran, Heather L.Parry, SharonCollings, PaulPartonen, TimoCollins, SamuelPasanen, TyttiCollins, LaPorchiaPaschalidou, Anastasia K.Colombo, ClaudioPaschall, MallieColson, GregoryPascual Aguilar, Juan AntonioComans, Tracy A.Pasini, DarioCommodore, AdwoaPassannante, MarianComte, KatiaPasti, LuisaConceição, Eusébio Z. E.Pastor, ManuelConcha, JeanniePatel, KushalConnelly, LukePatel, Neela K.Conrad, ZachPathak, Ram D.Conrad, AmyPatton, AllisonConte, AnnamariaPaudel, ShishirConti, SaraPaull, SaraContini, DanielePaulsen, PeterContini, DanielePaul-Ward, AmyContzen, NadjaPauly, VanessaConvertino, MatteoPavagadhi, ShrutiCooke, BarbaraPavic, AleksandarCooper, KayPavlopoulou, IoannaCooper, SimonPawlas, NataliaCope, Anna BarryPazdro, RobertCope, MichaelPaz-Ferreiro, JorgeCori, LilianaPeace, MichelleCornish, StephenPearce, Dora ClaireCorrea, Maria ElviraPearson, JamesCorrea Marques, RejanePeck, Richard M.Correa-Burrows, PaulinaPeckham, ErinCorsi, Daniel J.Pecurariu, Oana FalupCortés-Castell, ErnestoPeer, NasheetaCortese, Daniel K.Peerenboom, Peter BobCosco, Theodore DPeijnenburg, WillieCosma, AlinaPeller, JulieCosta, Nuno Manuel Sessarego Marques DaPelletier, GuillaumeCosta, PaulPeltier, RichardCote, Pierre-OlivierPeña Quintana, LuisCotton, Robin T.Peng, Szu-HsienCoulson, GuyPeng, Chien-fangCoulter, JohnPengo, MartinoCoussens, ScottPenkala, StefaniaCova, ThomasPennock, NathanCox, GeorginaPentakota, Sri RamCoxson, PamelaPereira, DoloresCoyne, MarkPereira-da-Silva, LuísCoyte, PeterPérez, PatricioCrabbe, HelenPérez, SandraCraig, LauraPérez Ballesta, PascualCraig, BrettPérez Castrillón, José LuisCramb, SusannaPérez-Rodrigo, CarmenCraparo, GiuseppePeri-Rotem, NitzanCraven, Michael P.Perlmuter, Lawrence CharlesCrema, StefanoPerrella, Sharon L.Criado-Alvarez, Juan JosePerry, LinCrisafi, FrancescaPerry, YaelCritchley, Hugo DPersson Waye, KerstinCroft, Rodney J.Perumal, DeepakCrohn, Helen M.Pes, Giovanni MarioCronin, AidanPetach, HelenCronkite, Ruth C.Peter, RichardCrooks, James L.Petermans, JeanCross, Paul C.Peters, Derek M.Crotty Alexander, LauraPeters, Ruud J. B.Crowley, JenniferPeters, RuthCrusius, JanPetersen, KristinaCryderman, DianaPetersen, EskildCsavina, JanaePeterson, Donna JeanCsiszar, AgnesPetrangeli Papini, MarcoCubadda, FrancescoPetrić, DušanCubero, Francisco JavierPetrie, Joshua G.Cucciniello, RaffaelePetroselli, AndreaCuller, CorinnaPetrusca, DanielaCummins, IanPetry, SebastianCunha, AngelaPettersson, JeanCunningham, Solveig ArgeseanuPetti, ClaudioCurl, Cynthia L.Pettinari, María LucreciaCurl, AngelaPetts, Richard J.Curran, LouisePfeffer, KarenCurtis, DenicePfoertner, Timo-KoljaČvorović, JelenaPhan, ThaiCycoń, MariuszPhibbs, Suzanne R.Cyrus, ElenaPhillips, CarlD’Elia, LanfrancoPhillips, KarenDa Silva Guerra, Danielle ReginaPhillips, JudithDąbrowska, JolantaPhillips, GregoryDalessandri, DomenicoPhongsavan, PhilayrathDaley, PeterPiao, YunxianDalmolin, RicardoPiatt, Gretchen A.Dalton, Alice M.Piffaretti, Jean-ClaudeD’Amico, MarioPignata, CristinaDancer, StephaniePignataro, GiuseppeD'Andrade, AmyPilcher, JuneDang, ZhiPillai, Vijayan K.Danielak, DorotaPineda, PalomaDaniell, WilliamPinnapireddy, Shashank ReddyDaniels, KevinPiotrowski, CarolineDaniels, JudithPiperakis, MichaelDanielson, Kirstie K.Piqueras Rodríguez, José AntonioDannenberg, AndrewPircher Verdorfer, ArminDantzler, PrentissPires, José Carlos MagalhãesDaoud, NihayaPirogova, ElenaDapprich, PeterPirrone, FedericaDas, AnshumanPistelli, FrancescoDasgupta, ShouroPistone, AlessandroDatta, RupaPithara, ChristallaDavid, AnnettePitt, JohnDavies, Anita A.Pitteri, MarcoDavies, EmmaPivonello, RosarioDavis, David E.Plain, Karren M.Davis, Claudia M.Planck, TerezaDavis, JamesPlano, DanielDawson, JohnPlumert, JodieDe Andrade, MarizaPochampally, Kishore K.De Blas, MaitePodgorski, David C.de Brito, JorgePoelman, Maartje P.De Cock, RozanePoglayen, GiovanniDe Donato, FrancescaPogson, MarkDe Francesco, EdiPokorn, MarkoDe Giglio, OsvaldaPolanska, KingaDe Graaff, FuusjePolen, TerryDe Gruijl, FrankPolesel, FabioDe Jesus Felicio, José AugustoPolidori, GuillaumeDe Jonge, JanPolk, DeborahDe Kwaadsteniet, LeontienPollack, KeshiaDe La Cruz Toledo, EliaPollitt, KrystalDe La Torre, Gabriel G.Polo-Kantola, PäiviDe Lourdes Pereira, MariaPolom, KarolDe Oliva, Samara UrbanPolya, DavidDe Oliveira Fernandes, EduardoPomatto, Mary CarolDe Palma, GiuseppePompili, MaurizioDe Souza, Roger-MarkPond, DimityDear, KeithPonikau, Jens U.Dearden, John C.Pontual, Emmanuel V.Dederichs, Anne SimonePopp, DennyDee, AnnePorsius, Jarry T.Deeprose, CatherinePortegijs, ErjaDeGrauw, XinyaoPortengen, LützenDeGuzman, Pamela B.Portnov, BorisDeierlein, AndreaPostnova, SvetlanaDeisenhammer, EberhardPottie, KevinDeJarnett, NatashaPoulas, KonstantinosDeKluizenaar, YvonnePoulsen, Dorthe VarningDel Giudice, RitaPounder, KieranDel Re, BrunellaPourchez, JérémieDeLauer, VernaPourret, OlivierDeLellis, NailyaPowell-Wiley, Tiffany M.Delgado, JordiPower, Thomas G.Delgado, MelvinPowers, James S.Delisle, HélènePowles, JohnDelmelle, Eric M.Prado, Gustavo FaibischewDelmonico, MatthewPrætorius, ThimDelong, GaylePratt, SarahDeng, PengPratt, Stephanie G.Deng, YongPredieri, GuerrinoDenham, Bryan E.Preece, Ellen P.Denholm, JustinPreethichandra, PreethiDening, TomPrensner, JohnDenny, ClarkPrice-Wolf, JenniferDentinho, TomazPrigent, AmélieDeo, RandhirPrince, LatrinaDepken, DianePrince, BennieDesai, Miraj U.Prior, Jerilynn C.Desai, JigarProcter, Sandra B.DeSesso, John M.Procter-Gray, ElizabethDeth, RichardProskocil, Becky J.Dettweiler, UlrichProta, FrancescoDeutsch, JonathanPrunuske, AmyDevane, Megan L.Pryss, RüdigerDevaney, CarmelPueyo Anchuela, ÓscarDevaraj, AishwaryaPujol, SophieDevilee, JeroenPulakka, AnnaDewan, AshrafPulido, Olga M.DeWeese, RobinPulignani, SilviaDewey, CatePulster, ErinDewhirst, TimothyPunshon, TracyDeWitt, Jamie C.Purcell, NinfaDey, AditiPurmehdi, MostafaDeymier, Alix C.Puy, JaumeDeziel, NicolePuzon, Geoffrey J.Dhana, KlodianPuzzolo, ElisaDhar, ParamitaPyke, ChrisDhuffar, ManpreetPype, Marie-LaureDi, JiangliQian, WuyongDi Ciaula, AgostinoQian, QinDi Cristo, CristianaQin, BoDi Domenico, Enea GinoQin, QuandeDi Liegro, ItaliaQin, KaiDi Maria, FrancescoQiu, RuofengDi Martino, FerdinandoQIU, JianyingDi Palma, LucaQu, ShaojianDias, SoniaQuadros, Patricia Dörr DeDias, Rolando C. S.Quarmby, TomDiaz, JulioQuevedo, LluisaDiaz, DuarteQuinlan, Jennifer J.Dickey, BretQuinn, AshlinnDiderichsen, FinnQuisenberry, Amanda J.Dieris-Hirche, JanQuock, RyanDiffin, JanetRabinowitz, SamuelDifranza, Joseph R.Racine, ElizabethDiller, KennethRada, Elena CristinaDillon-Wallace, Julie A.Radziemska, MajaDimova, ElitsaRafaj, PeterDing, QiangRagas, Ad M. J.Ding, TianbingRage, EstelleDini, LucianaRahman, RahbelDinkel, DanaeRahman, AzizDiraco, GiovanniRajaee, MozhgonDirk, RichterRajeev, SreekumariDirks, KimRajurkar, MihirDiroll, BenjaminRakshit, SudiptaDizmen, CoskunRalf, RisserDjebou, Dagbegnon C. SohoulandeRalston, MargaretDluholucký, SvetozárRalston, Margaret L.Do, ThuyRamakrishnan, SreejithDobson, MarnieRamanathan, SubhaDockery, DouglasRamirez, MarizenDodds, W. JeanRamírez-Santana, MurielDoerr, CelesteRamos, PatriciaDolney, Timothy J.Ramos, AthenaDomek, Gretchen J.Ramos Miras, Jose JoaquinDomingo, RosarioRamsahoye, BernardDomingue, MichaelRamsay, Michael A.Donat Vargas, CarolinaRamsden, VivianDonati, CristianaRamström, Lars M.Donatuto, JamieRana, SatianderDong, ZhaominRana, DipakDong, ZhaoRanabhat, ChhabiDonny, Eric C.Ranadheera, SenakaDonohue, MauraRand, MatthewDonovan, John E.Randall-Simpson, JanisDonzelli, GabrieleRanjitkar, SarbinDorea, Caetano C.Ranmuthugala, GeethaDorosh, AndriyRantakokko, MerjaDorri, MojtabaRantanen, VilleDorszewski, PiotrRao, SheelaDortet, LaurentRapp, KilianDos Santos, Eduardo RibeiroRashedi, EhsanDoshi, SaumilRasul, AzadDou, ZhichengRato, Luís Pedro FerreiraDougherty, Michael JohnRatschen, ElenaDowning, StevenRaven, MelissaDoyle, FrankRaynes-Greenow, CamilleDrago, FrancescoRayson, Gary D.Drancourt, MichelRedding, ColleenDrapeau, ChristopherReddy, Ravi KrishnanDreassi, EmanuelaReddy, SandeepDrews-Botsch, CarolynRediske, Richard R.Driezen, PeteReece, LindseyDritselis, ChrisReed, DerekDroste, DirkReed, Edward JustyDroste, NicReeves, AaronDrottz-Sjöberg, Britt-MarieRege, ShraddhaDrzazga, ZofiaRehkopf, DavidDu, ShushanRehman, Junaid U.Duan, HanchenRehman, LaureneDubois, UteReichmayr, JohannesDuby, ZoeReifsnider, ElizabethDueker, M. EliasReinhardt, Jan DietrichDugue, BenoitReis, StefanDumbili, EmekaReisen, William K.Dummer, TrevorReisi, MarziehDunkle, Ruth E.Reiter, E. MirandaDunne, Eileen M.Ren, QingzhongDunstan, DavidRen, YanfangDuodu, GodfredRen, JinmaDupras, CharlesRengasamy, SamyDuquette, PierreRenzetti, Claire M.Duran, NelidaRequia, Weeberb J.Durando, PaoloRéquia, WeeberbDurand-Zaleski, IsabelleResch, FranzDurkin, Anne E.Reszka, EdytaDuron, JacquelynnReutfors, JohanDuszynska, WieslawaRey, JorgeDwivedi, DipankarReyes, JeanetteDyche, JeffReynolds, AmyDyer, TomReynolds, LoriDyson, Yarneccia D.Rhoades, MarthaEastin, Matthew D.Ribeiro, Ana Isabel CorreiaEbeling, PeterRibeiro, HelenaEbersviller, SethRibeiro, Ana IsabelEbisu, KeitaRibeiro, EdnaEbmeier, KlausRicceri, FulvioEcheverri, MargaritaRiccio, PaoloEckel, Sandrah P.Riccio, AngeloEckert, StineRiccio, GiuseppeEddyani, MiriamRice, Penelope A.Edgar, DaleRice, Robert H.Edwards, Joshua R.Rice, MarkEedunuri, Vijay KumarRichards, LauraEfird, JimmyRichardson, CliveEgashira, ShinjiRichmond, JanetEgawa, ShinichiRichter, KneginjaEguchi, NawoRiedel, NatalieEhlers, Lars HolgerRiediger, Natalie D.Ehlis, Ann-ChristineRietra, ReneEhrich, MarionRigby, PatriciaEichstaedt, ChristinaRiggs, ElishaEid, PatriciaRigotti, ThomasEime, Rochelle M.Riiser, AmundEiroa-Orosa, Francisco JoseRikard, R. V.Eisler, KarenRiley, Karin L.Ekblad, MikaelRiley, Lee W.Ekmekcioglu, CemRimondini, LiaEkström, Eva Charlotte M.Riquelme, AdriánEkundayo, OlugbemigaRiveron, JacobEkúndayò, Olúgbémiga T.Robbins, Miriam R.Ekuni, DaisukeRoberts, Jennifer D.Ekwue, EdwinRobichaud, AlainEl Ghoch, MarwanRobins, AlanEl Khalloufi, FatimaRobinson, LisaEl Shahawy, OmarRobles, LukeEldridge, Alison L.Rocklöv, JoacimEleftheriadis, TheodorosRocklöv, Åsa HolmnerElgazzar, RedaRöder, StefanEliecer Coto, Eliecer CotoRoderick, PaulElinder, Carl-GustafRodgers, Rachel F.Elk, RonitRodrigo-Comino, JesúsElliott, MarkRodrigues, Jean-MarieElliott, LewisRodríguez, Jesús ÁlvarezElliott, Michael B.Rodriguez-Rodriguez, FernandoElmenhorst, Eva-MariaRodríguez-Seijo, AndresElstad, Jon IvarRodziewicz, JoannaElvik, RuneRoebuck, BillEmmanuel, EvensRoest, KeesEmond, Jennifer A.Roex, ErwinEngberg, Sandra J.Rogers, ToddEngler-Stringer, RachelRogerson, MikeErazo, MarciaRohde, NicholasErbacher, Terri A.Rojas-Rueda, DavidErbas, BircanRomani, MassimoErikson, KeithRomano, MeganEriksson, CharliRomano, NiclaEronen, JohannaRomano, AudreyErrede, DawnRomano, MeganErreygers, GuidoRomanowski, Kathleen S.Errington, GailRomer, DanEsmene, ShukruRomero, InmaculadaEsparcia, JavierRoom, RobinEspinel-Ingroff, AnaRoot, Martin M.Espinosa, Hugo G.Rosa, Maria JoséEspinosa-Ortiz, ErikaRosário, RafaelaEsposito, CiroRosenbluth, Jennifer M.Essen, Elisabeth VonRoss, VeerleEstape, Estela S.Rossi, Paolo GiorgiEvans, MaryRossi, René M.Evelyn, TalbottRostaing, LionelEvens, AnneRoswall, NinaEverett, KevinRott, EduardEverts-Panman, ChantalRoumpakis, AntoniosEwen, Heidi HRovira, PereExadaktylos, AristomenisRowlinson, SteveEzedine, BouhlelRoy, DenisFabbricatore, MariantoniettaRóżalska, BarbaraFabbricino, MassimilianoRozsival, PavelFabiano, BrunoRuano Ravina, AlbertoFabregat, AzaelRubinelli, SaraFactor-Litvak, PamRucci, PaolaFairlie, IanRüegg, JoelleFajersztajn, LaisRuffalo, LeslieFalandysz, JerzyRuiz-Canela, MiguelFalciglia, Pietro P.Ruiz-García, AlejandroFalconer, IanRuiz-Narváez, Edward A.Falk, MartinRuiz-Robledillo, NicolásFalkenstein, MichaelRummo, PasqualeFalkinham, JosephRuscica, MassimilianoFan, TijunRussell, JoannaFan, ChaoRussell, MarieFan, Wen-HongRussell, ArmisteadFan, BinRussell, Fraser D.Fang, ZhengRust, MichaelFaraj, JoycelynRüttiger, LukasFarber, Naomi B.Ryabov, IgorFarhi, DollyRyan, MichaelFarina, NicolasRyder, SheilaFarma, Jeffrey M.Rydstedt, LeifFarooq, MuhammadRyser, ElliotFarrell, Anne F.Saad, MohamadFarrington, TomSacande, MoctarFasano, EvelinaSachmechi, IssacFauchier, Angele C.Sacristán, DanielFaustini, AnnunziataSádaba, CharoFavas, PauloSadat, JasminFei, Andrew Chang-YoungSaddik, BasemaFeingold, Beth J.Sadek, Hesham A.Feng, YuSadler, EuanFeng, LeiSaez, MarcFenton, SueSafavi, KamranFera, MarcelloSagebiel, John C.Ferguson, AlesiaSaha, SanjibFernandez, ElizabethSaha, ShubhayuFernández, Julio BlancoSainani, KristinFernández-Bustos, Juan GregorioSainz, CarlosFerrara, NicolaSaito, KanFerrari, EdSajobi, TolulopeFerrario, Marco M.Sakai, ToshioFerras, Luís Jorge LimaSakakibara, MasayukiFerrero, EnricoSakan, Sanja M.Ferro, Giancarlo AntonioSakata, MasahiroFiaschi, SimoneSakellariou, DikaiosFigueiredo, RafaelSakhaee, KhashayarFigwer, JarosławSaksena, SumeetFilon, Francesca LareseSakumura, YuichiFine, James BurkeSala, RoserFinka, AndrijaSalahudeen, MohammedFinkel, MadelonSalaris, LuisaFinlay-Jones, AmySalas, Lucas A.Finn, SymmaSalazar, AlejandroFiocchi, SerenaSalazar, Natalia VillotaFiorito, SilvanaSalazar, Leslie RamosFirstenberg, MichaelSalazar, Debra J.Fischer, FlorianSalgado, Cassandra D.Fischer, GabrieleSallam, Mohamed F.Fischl, Andrea F. R.Salm, Adrienne K.Fish, JulieSalmon, Charles ThomasFisher, Gwenith G.Salom, ScottFitzgerald, NeilSalter, Linda-RuthFitzgerald, NiamhSamadi, Sayyed AliFitzsimmons, SuzanneSambamoorthi, UshaFlanagan, Sara V.Sambri, VittorioFlapper, Simme DouweSammartino, Maria PiaFlecha, RamonSampson, Natalie R.Fleischhacker, SheilaSan Jose, RobertoFleiter, JudySandbæk, MonaFleming, Richard K.Sandra, JaksicFletcher, Barbara A. SworeSandy, LarissaFlorek, EwaSankaranarayanan, Nehru VijiFlorence, Curtis S.Sankavaram, KavithaFloros, GeorgiosSanmiquel Pera, LluisFluegge, KyleSano, DaisukeFlynn, JenniferSantamaria, RitaFoletta, Victoria C.Santarelli, LoryFonseca, Ana SofiaSantikarn, ChamaiparnFont, GuillerminaSantilio, AngelaFontanals, NúriaSantos, Maria Teresa L. DosFoody, MairéadSantos, Maria JacintaFoong, Rachel E.Santos, Maria PaulaFoos, BrendaSantos Zanchetta, MargarethFoote, JulieSantos-Gallego, CarlosFord, DawnSardaro, RuggieroFord, JudySargentis, George-FivosForjuoh, Samuel N.Sarkar, SumitForst, LindaSarpong, Daniel F.Forward, SonjaSasseville, DenisFoster, TimSatchidanand, NikhilFoster, LaurenSaunders, Daniel G.Fouladkhah, AliyarSaurina, CarmeFounda, DimitraSavelyev, AlexanderFoust, Richard D.Savoie Roskos, MatejaFox, MarySawa, TeijiFox, GlenSbihi, HindFrade, JoãoScannell Bryan, MollyFragkos, KonstantinosScasny, MilanFram, Maryah StellaSchaffner, DonaldFrancescutti, LouisSchalinski, IngaFrancis, GarySchechter, Julia CorwinFrancis, JoelScheepers, Paul T. J.Franck, UlrichSchembari, AnnaFrank, Janet C.Schepelmann, PhilippFrank, KevinScherr, SebastianFrank, SašaSchieweck, AlexandraFranklin, PeterSchiff, MiriamFranz, EelcoSchilaty, NathanFranzen, DaveSchimmenti, AdrianoFranzese, AdrianaSchindler, StefanFrayne, BruceSchiza, Sophia E.Frazer, KateSchmalwieser, Alois W.Frederick, ArmahSchmeling, AndreasFreedman, Darcy A.Schmeltz, Michael T.Freeman, TobySchmidt, MichaelFreeman, ShrirraSchmidt, AmandFreihaut, James D.Schmidt, ChristinaFrew, Paula M.Schmidt, SilkeFrey, LauraSchmitt, Laetitia Helene MarieFried, Eiko I.Schneeweis, AdinaFriedman, S. H.Schneider, PetraFriedrich, RainerSchneider, AlexandraFriger, MichaelSchnell, IzhakFristedt, SofiSchoenhagen, PaulFroehlich-Grobe, KatherineSchomer, PaulFróes, CarmenSchonthal, AxelFröhlich, EleonoreSchöpfer, JuttaFrohne, TinaSchreiner, PamelaFronczyk, JoannaSchroeckh, VolkerFrugé, AndrewSchroeder, Megan JFrumkin, HowardSchroeder, WinfriedFtanou, MariaSchubert, SebastianFu, QiangSchubert, JochenFuchs, Judith A.Schullehner, JörgFudge, NinaSchultz, AndreFuente, VicentaSchulz, Peter J.Fuentes-Bargues, José LuisSchulze, Matthias B.Fuertes, ElaineSchumann, BarbaraFujibe, FumiakiSchutte, AnneFujii, YasuhitoSchwab, KeriFujita, MasayukiSchwartz, GaryFuks, KaterynaSchwartz, BrianFukuda, YoshiharuSchwarz, EliFukuda, MisaoSchweitzer, DietrichFuller, RichardScortichini, MatteoFulthorpe, RobertaScott, PippaFung, Jimmy C. H.Scott, BritainFuruhashi, KazukiScott, StephanieFuruta, MichikoScott, IainFuster, HéctorScotti, MarcusGabbay, MarkScrine, ClairGabutti, GiovanniSeaman, KateGadagbui, BernardSears, LouisGadomski, Anne M.Seckmeyer, GuntherGaffo, Angelo L.Secondi, LucaGalanis, AthanasiosSeebauer, SebastianGalante, FrancescoSeeman, Mary V.Galinha, CatarinaSegura-Ortí, EvaGallelli, LucaSeidel, MichaelGallis, ChristosSeixas, AdéritoGamaldo, AlyssaSekine, YoshikaGamble, Donald S.Sell, KatieGamet-Payrastre, LaurenceSelland, CoreyGander, JenniferSellarés, JacobGandre, CoralieSellix, Michael T.Gannon, JudieSelvamani, AmuthaGao, ZhaohuiSelya, ArielleGao, XiangpengSembajwe, GraceGao, MengSemmens, ErinGarcia, ValerieSera, FrancescoGarcia, João Nuno Pinto MirandaSerafini, GianlucaGarcia, JavierSerpico, RosarioGarcía, FernandoSerrão, José EduardoGarcia Bravo, AndreaServadio, PierannaGarcía-Bermejo, JuanServidio, RoccoGarcía-Matamoros, Jorge BediaSeth, DevanshiGarcía-Pérez, JavierSetti, IlariaGardner, Paul D.Setyan, AriGardner, MarkSeverine, DeguenGarfield, SaraSha, ZheGari, MerceShaban, Ramon Z.Garnham, BridgetShah, TayyabGartner, CoralShahabfar, AlirezaGary-Webb, TiffanyShahid, SaroashGarza-Rodríguez, Iliana De LaShahid, RizwanGasana, JanvierShahmoradi, MahdiGašević, DanijelaShaker, RichardGasparatos, DionisiosShamasunder, BhavnaGast, RebeccaShang, CeGatersleben, BirgittaShankar, AparnaGay, MelvinShanmugam, MuruganandanGaynor, KeithShao, ZhenfengGazzola, PatriziaSharan, RitiGdula-Argasińska, JoannaShareck, MartineGe, ErjiaSharma, RohitGe, CuiShaw, RajibGe, XiaodongShaw, Richard J.Gea, Oliveri ContiSheehan, DiannaGebbie, KristineSheikh, Sophia S.Gee, SarahShelton, NicolaGeidne, SusannaShen, JiabinGeipel, GerhardShen, JunGeisler, CorinnaShen, ZhenjiangGemar, MasonShen, SuwanGennari, FrancescaSheppard, PaulaGennuso, Keith P.Sheppard, Amanda J.Geores, MarthaSherchan, SamendraGeorgiadis, PatroklosSherratt, FredGerard, F. JeaneShewale, SwapnilGerger, HeikeShi, BaoyouGerrits, LasseShi, VictorGerry, ChristopherShi, YuanGhaffar, SeyedShi, YuyanGhahari, SetarehShi, HuijingGhanouni, AlexShiau, Tzay-AnGhinea, Razvan IonutShih, Patrick C.Ghosh-Dastidar, BonnieShil, NirajGiaccone, ValerioShim, JaejunGiacomino, AgneseShim, Sang-OhGiammanco, AnnaShimek, Jo AnnaGianatsi, MyrsiniShin, Won SopGibb, HermanShin, Dong-chunGibbs, Bernhard F.Shin, DayeonGibson, LauraShishegar, NastaranGiedych, RenataShoffstall, Andrew J.Gifford, SandraShoji, TsuchidaGilden, RobynShoji, KotaroGiles, Luisa V.Shoji, KensukeGiles, AudreyShook, AllanGilissen, RenskeShyr, Oliver F.Gill, TiffanySi, LeiGill, Simone V.Sicard, PierreGillander-Gådin, KatjaSiceloff, E. RebekahGillespie, DuncanSiceloff, RebekahGilliver, MeganSicotte, DianeGilmour, StuartSiddall, AndyGingrich, ChrisSieber, NancyGiorgi, GabrieleSiega-Riz, Anna MariaGiovene Di Girasole, EleonoraSiegel, RobertGiraldo, FaberSienkiewicz, ZenonGisbert, Javier P.Sigler, JeffreyGitau, MargaretSignorelli, Salvatore SantoGiuliani, DiegoSigurvinsdottir, Rannveig S.Giuliano, MarinaSilfee, ValerieGłąbska, DominikaSillanpää, MikaGlass, JosephSilva, Gabriela JorgeGlicksman, AllenSilva, LígiaGlynn, Peter J.Silva, Cristiano M.Gnambs, TimoSilvan, Jose ManuelGobbi, EricaSim, KyraGobejishvili, LeilaSim, Chang SunGodar, Dianne E.Simmering, Jacob E.Goettel, MarkSimões, Marta FilipaGogos, CharalambosSimon, Marie-ChristineGoicoa, TomásSimonds, VanessaGolasi, IacopoSimons, Shellie R.Gold, Laura S.Simplican, Stacy CliffordGoldstein, Adam O.Sims, JenniferGoletti, DeliaSims, JaneGolicnik, BarbaraSinclair, RyanGolla, VijaySinden, KathrynGoltz, Douglas M.Sindi, ShireenGombac, ValentinaSinger, Andrew C.Gomes, Ana P.Singh, Atul KumarGómez Barrios, XiomarSingh, HarsimranGómez Piqueras, PedroSingh, SanjayGómez-Bezares, FernandoSingh, NeenuGómez-Losada, AlvaroSingleton, ChelseaGonçalves, LuziaSingovszka, EvaGong, YangSiniscalco, DarioGonzalez-Barcala, Francisco JavierSioris, ChristopherGonzalez-Casanova, InesSiraj, Amir S.Goodman, JulieSirca, CostantinoGoodman, PatŠirić, IvanGoodwin, MichaelaSirsat, SujataGoodwin, DeniseSit, CindyGoodwin, Kelly D.Sivaram, SudhaGoodwin, IanSjödin, FredrikGopalakrishnan, BhaskaranSkärbäck, ErikGopal-Kothandapani, Jaya SujathaSkelin, Ana KuzmanićGorczynski, PaulSkoczko, IwonaGordon, RossSkouloudis, Andreas N.Gordon, Derrick M.Skrzypiec, GraceGörlich, DennisSkwarzec, BogdanGou, ZhonghuaSlack, FrancesGoulas, AntonisSlaughter, JamesGoyal, RajniSloan, ChantelGraber, JudithSloan, Robert AlanGrabich, ShannonSlot, Jason C.Grady, SueSmarr, MelissaGraffelman, JanSmeets, Patrick W. M. H.Granada, Camille E.Smit, Brandon W.Grandner, Michael A.Smith, ThomasGranja, MónicaSmith, StuartGrant, WilliamSmith, Leonard B.Grant Ludwig, LisaSmith, AndyGrases, FelixSmith, Brittany L.Grattan, Lynn M.Smith, David I.Graveling, RichardSmith, Ryan C.Graves, LeeSmith, Julie A.Gray, LesleySmith, Genee S.Gray, DeborahSmith, Miichael (Mike)Gray, EmilieSmythe, DavidGray, AndrewSnijders, DeborahGreen, James A.Snitker, SorenGreen, NathanSnyder, EricGreenberg, ArthurSoares, PaulaGreenleaf, Arie T.Sobota, AleksanderGreer, Anna E.Socol, YehoshuaGreim, HelmutSohaib, OsamaGrekousis, GeorgeSoiza, Roy L.Greppi, AnnaSojic, AleksandraGrether-Beck, SusanneSokal-Gutierrez, KarenGrieger, Jessica A.Soles, GillianGriffin, BronwynSolo-Gabriele, HelenaGrifoni, Roberta CocciSolomon, PattyGrigsby-Toussaint, Diana S.Solovyev, NikolayGrischek, ThomasSommer, HaraldGrochans, ElzbietaSone, HirohitoGroffik, DorotaSone, HidekoGrossman, EllieSong, FujianGrucza, RichardSong, GuoGrunig, GabrieleSong, RanranGrunseit, AnneSong, JinxiGrzybowska, KatarzynaSoos, IstvanGrzybowska, EwaSornay-Rendu, ElisabethGu, DananSoudeyns, HugoGu, YuanyuanSoukupová, JanaGuerriero, MassimoSoulage, ChristopheGugusheff, JessicaSoultos, NikolaosGuilcher, SaraSoundy, AndyGuillén, J.Sousa, Carla A.Guina, JeffreySousa, Joao CarlosGuitton, Matthieu J.Sousa, NelsonGulati, MeghaSousa Fragoso, RuiGulliver, PaulineSouthworth, MichaelGullón Tosio, PedroSowa-Kofta, AgnieszkaGulson, BrianSpangler, JohnGumber, AnilSpear, Suzanne E.Gumbo, Jabulani R.Spencer-Hwang, RhondaGun, RichardSperlich, BirgitGundogdu, OzanSpieckermann, SvenGunier, BobSpilanis, IoannisGunnarsdottir, IngibjorgSpinazzè, AndreaGuo, YuSpires, StevenGuo, Chun XiangSpitz, LewisGusiatin, MariuszSpörndly, RolfGustafson, AlisonSpringgate, BenGustafsson, PeikSpüler, MartinGuthrie, RobertSt Clair, Michelle C.Gutierrez, KristieSt John, James A.Gütschow, JohannesSt. Hilaire, Melissa A.Guzek, DominikaSt.Helen, GideonGuziana, BozenaStabile, LucaGuzzi, GianpaoloStacey, BrandonGyorkos, ChristinaStack, StevenHa, HoehunStadtlander, TimoHa, SandieStafoggia, MassimoHaapala, Eero A.Stagnitti, KarenHaarbauer-Krupa, JulietStagos, DimitriosHaase, HajoStahl Jr., Ralph G.Haboubi, NadimStaley, Zachery R.Habuda-Stanic, MirnaStallings-Smith, SericeaHachiya, TsuyoshiStalsberg, HelgeHackett, RuthStam, RianneHadimani, Ravi L.Stamatis, NikolaosHadjikakou, Sotiris K.Stamey, James D.Hagan, ChristopherStanca, Sarmiza ElenaHagen-Zanker, AlexStanek, AgataHagiwara, NaoStanišić Stojić, SvetlanaHagner-Derengowska, MagdalenaStanistreet, DebbiHaile, Tadele MeashoStanistreet, DebbiHailer, M. KatieStark, EmilyHajek, AndréStarr, Martha A.Hajj, DanaStathis, DimitriosHakulinen, ChristianStauvermann, Peter JosefHales, HeidiStavi, IlanHalik, ÜmütStavropoulos-Kalinoglou, AntoniosHall, NathanielStearns, Jodie A.Hall, MichaelStearns, DianeHall, SophieStedman-Smith, MaggieHall, RalphSteenkamp, MalindaHall, H. IreneSteeves, Betsy AndersonHall-Clark, BrittanyStefanakis, AlexandrosHalonen, Jaana I.Stehlik, DanielaHalsall, JamieStehr, MarkHalsband, ClaudiaStein, Robert M.Haluza, DanielaStelmach-Mardas, MartaHamann, MartineStephens, AlexandreHamano, TsuyoshiStermac, LanaHamat, Rukman AwangStevens, DavidHamer, GabrielStevinson, ClareHametner, BernhardStewart, Anna M.Hamilton, DavidStewart, Alan E.Hamilton-Williams, EmmaStewart, Barclay T.Han, Young-JiSticca, FabioHan, Dong WookStiehl, EmilyHan, XuemeiStilianakis, Nikolaos I.Han, GangStillman, MartinHan, QifengStingone, JeanetteHan, Seung JinStins, John F.Han, Seung-YongStivrins, NormundsHan, BingStockings, EmilyHaney, JenniferStoehr, TobiasHang, XiaoshuaiStoforos, Nikolaos G.Hanke, WojciechStoltz, JonathanHanley, JanetStolz, ErwinHanly, Paul A.Stone, AndreaHansen, MargaretStoneham, MelissaHansen, AlanaStoner, LeeHansen, Anders Blædel GottliebStortini, Angela MariaHaque, ReinaStout, David A.Haque, SairaStrady, EmilieHarada, KazuhiroStrong, CarolHarada, Kouji H.Strubelt, HenningHarbison, JustinStylos, NikolaosHarding, IanSu, Hsin-TingHariharan, HarrySu, ShiliangHaring, RobinSu, ShaoyongHarlan, SharonSu, Yuan-FongHarrell, PaulSubbiah, MuruganHarrington, Kathleen F.Subhan, Fatheema BegumHarris, Allyssa L.Subramaniam, PrasadHarris, J. IreneSubramanian, DevikaHarris, KeithSudano, Laura ElizabethHarrison, Sayward E.Sueki, HajimeHarry, KambezidisSuen, Sze-chuanHarstad, IngunnSugg, Maggie M.Hart, Joy L.Suggs, SuzanneHarting, JannekeSugiyam, KojiHartmann, James X.Sugiyama, TakemiHarvey, CarolSuh, Dong HeeHarvey, AdamSuhaili Ramli, NurHarville, EmilySukumaran, AnilHashimoto, NaotoSullivan, Elinor L.Hassan, RohailSullivan, MarianneHatfield, JerrySumner, WaltonHattaf, KhalidSun, QuanfuHatzopoulos, StavrosSun, SunHatzopoulou, MarianneSun, LingHauser, PeterSun, DianjianyiHawcroft, ClaireSun, Li-YunHawes, MarthaSun, WeilingHawkins, Roxanne D.Sun, ZhifuHawkins, KeelySun, WenjieHawkins, MarquisSundaram, RajeshwariHawley, BrieSuñer Soler, Maria RosaHayami, HitoshiSung, JidongHayes, RichardSuo, WeilanHayman, Laura L.Sureda, FranciscaHaynes-Maslow, LindseySussan, ThomasHayward, R. DavidSutaria, DhruvitkumarHazelton, PamelaSutphin, SuzanneHe, DaihaiSutton, JeannetteHe, YiSuurmond, JeanineHe, HongxuanSuwazono, YasushiHe, GuojianSvanemyr, JoarHe, Qi-QiangSvartengren, MagnusHe, XiaoqingSvensson, JannetHe, QiangSvob, ConnieHealy, KristenSwann, Brian JeffreyHeaton, Matthew J.Swanson, JohnHeffelfinger, JamesSwenson, Cynthia CupitHeffner, Ashley LewisŚwiątkowska, BeataHeft, HarrySy, IbrahimaHegerl, UlrichSygit, KatarzynaHeidmann, IrisSymonds, Erin M.Heinen, MirjamSymons, MartynHeinonen-Tanski, HelviSzafron, MichaelHejmanowski, RyszardSzekely, GyorgyHelbich, MarcoSzékely, AndreaHellwig, MichaelSzulczewska, BarbaraHelm, PaulSzymańska-Pulikowska, AgataHemphill, Jean CroceSzyszkowicz, MieczysławHenderson, BarronTadić, VanjaHendricks, Joan C.Takada, SatoruHendriksz, Christian J.Takahashi, KenHendryx, MichaelTakahashi, HajimeHenning, Johannes I. F.Takahisa, KanekiyoHenninger, William R.Takala, JukkaHenning-Smith, CarrieTakamura, NoboruHenrickson, MarkTakano, TomomiHenry, TiernanTakebayashi, HidekiHenry, KevinTakebayashi, ShigeoHerbert, RobTaliaferro, LindsayHerlache-Pretzer, EllenTall, Ben D.Hernández, FranciscaTammelin, TuijaHerr, Raphael M.Tamosaitiene, JolantaHerranz, Raul CanoTamulevicius, NaurisHerrera, RonaldTamura, AtsutakaHerrera, ManuelTan, Chun LiangHerrera-Melián, José AlbertoTanabe, MasakiHerrmann, AlinaTang, XiaochenHerrmann, FabianTang, JianjunHerzog, KarinTang, RobertHerzog, HaroldTang, YilangHess, Catherine AnnTang, NaijunHeßling, MartinTang, Meng-ChiHidaka, Brandon H.Tanigawa, KoichiHigginbotham, NickTanner, GritHiilamo, HeikkiTanton, RobertHiller, SusanTao, ShashaHillier, AmyTao, YanHilton, ClaudiaTaplin, StephanieHimmelstein, MaryTarabieh, KhaledHimmerich, HubertusTarazona-Santabalbina, Francisco José OséHinojosa, Melanie SbernaTaren, DouglasHinwood, AndreaTargiel, KrzysztofHinyard, Leslie J.Tarlov, ElizabethHirai, ItaruTarnoki, Adam DomonkosHiraishi, AkiraTartakovsky, LeonidHirata, AkimasaTasmin, SairaHirt, Edward R.Tavernier, JanHirvensalo, MirjaTavori, HagaiHiscock, RosemaryTawia, SusanHitchings, RussellTaylor, GrahamHjorth, PederTaylor, Cristopher M.Ho, Roger Chun-ManTaylor-Robinson, Andrew W.Ho, ChinYuTchangani, Ayeley P.Hoagwood, KimberlyTe, Shu HarnHoare, ErinTecles, FernandoHock, EmmaTefft, NathanHodge, AllisonTeismann, TobiasHodgson, IanTeixeira, SamanthaHoekstra, TrynkeTelfair, JosephHoepner, LoriTeli, DespoinaHoffer Gittell, JodyTempera, ItaloHoffmann, FalkTemple, NormanHofman, JelleTempleton, MichaelHogan, AnthonyTenkate, ThomasHogg-Johnson, SheilahTeras, Lauren R.Hollar, David W.Terblanche, LourieHolle, RolfTerry, DanielHolloway, Ian W.Tervonen, HannaHolloway, Alison C.Teske, JenniferHolzgraefe, BernhardTesler, RikiHonda, AkikoTestoni, InesHondula, DavidTetrick, Lois EHong, PeiyingThai, PhongHong, Young-SeoubThanavala, YasminHönig, VáclavThapa, SubashHoover, Joseph H.Theodorsson, Gudjon ElvarHori, YuuichiTheorell, TöresHori, HajimeTheorell-Haglöw, JennyHoriuchi, MasahiroThiara, RaviHoriuchi, SatoshiThibodeau, AlexandreHorst, JeremyThibodeaux, TerryHorton, Teresa H.Thiel, Cassandra L.Horton, GraemeThielemann, ChristianeHorvai, GeorgeThielens, ArnoHoshiko, SumiThigpen, CalvinHossein-Nezhad, ArashThikkurissy, SaratHoughton, KMThøgersen-Ntoumani, CecilieHovmand, Peter S.Thoma, MyriamHoward, Annie GreenThomas, Roger E.Howard Wilsher, StephanieThomas, KevinHowarth, MichelleThomas, Stephen B.Howell, Andrew J.Thomas, Elizabeth A.Hribar, LawrenceThompson, WendyHseu, Zeng-YeiThompson, AndrewHsia, Shih-MinThorsteinsson, EinarHsieh, Cheng-HsiungThrane, UlfHsieh, Yi-PingThunders, MichelleHsu, Hui-ChuanThurber, MarkHsu, StephenThuy, La Thi ThanhHsu, Hui-ChuanTian, YilinHsu, MinnaTiefelsdorf, MichaelHsu, Ming-FuTiehm, AndreasHu, HuiTigbe, WilliamHu, GuoqingTimmermans, LucHu, BoTimmermans, HarryHu, ZhiyongTimmins, KateHuang, Yu-TungTin Tin, SandarHuang, ChaoTinnerberg, HåkanHuang, Yu-FangTippett, VivienneHuang, Wu-JangToccolini, AlessandroHuang, Hung-ChungTochkov, KirilHuang, HaoToderi, StefanoHuang, GuangqunTogo, FumiharuHuang, ZhongweiToivanen, SusannaHuang, YikunTokoro, ChiharuHuang, XiaoTomaselli, VeneraHuber, JörgTomášková, HanaHuettmann, FalkTomaszewska, EwaHughey, MorganTomata, YasutakeHuguet, NathalieTomczak, AndrzejHui, XuanTomioka, MichiyoHülür, GizemTommasino, LuigiHume, Kelly R.Ton, Shan-ShinHummel, KarinTong, LiangHung, Pei-YinTong, ZhemingHung, Ivan F. N.Tong, ZhijunHung, Yung-TseTong, Hoang V.Hung, Mei-ChuanToniolo, Antonio Q.Hung, Galen Chin-LunToomey, ClodaghHunt, PamelaTorio, Celeste MarieHunt, MercedesToropova, AllaHunter, RuthTorres, ElisaHursthouse, AndrewTorres, João PauloHurtig Wennlöf, AnitaTorres De Oliviera, RuiHusáková, LenkaTorres Lagares, DanielHuss, FredrikTorres López, Mª IsabelHutchinson, JayneTorres-Barceló, ClaraHutton, VickiTorres-Padrón, M. EstherHyde, MelissaToscano, WilliamIan, PaganoToumba, MeropiIanieri, AdrianaTountas, YannisIckes, MelindaTowne, Samuel D.Idler, ChristineTownsend, KristyIfelebuegu, AugustineTran, HuyIgic, IvanaTrawley, StevenIgnatowicz, KatarzynaTrevisan, AndreaIguchi, TaisenTrinh, LindaIhle, AndreasTrovato, Francesca MariaIijima, ShigeoTrudeau, FrancoisImeson, AntonTrue, LarissaImperiale, DavideTrulson, Chad R.Infante-Rivard, ClaireTsagarakis, KonstantinosIngusci, EmanuelaTsai, Chen-ChiInniss, Enos C.Tsai, Jing-JaneInnocent, GilesTsai, Li-TangInohara, TakehiroTsao, Lee-IngInoue, IkuoTsapakis, IoannisInoue, Ken-ichiroTseles, DimitriosIoka, SeiichiroTseng, Yi-PingIqbal, Tariq H.Tseng, Tung-SungIryo-Asano, MihoTseng, Chun-MaoIsaacson, MichalTseng, Hsu-MinIsani, GloriaTsiamis, CostasIsgor, ZeynepTsikouras, PanagiotisIsidoro, Jorge M. G. P.Tsionas, Mike G.Islam, Mir RabiulTsitsos, WilliamIslam, RafiqTso, For YueIsmail, AhmedTsourtos, GeorgeIstvan, HernadiTsow, Francis (Tsing)Ittenbach, Richard F.Tsugane, ShoichiroIuliano, SandraTucker, JohnIvanov, Alexander V.Tume, PedroIvey, CesunicaTuomisto, JoukoIvey, Cesunica E.Turenne, Christine Y.Iwahori, ToshiyukiTurgeon, NathalieIzenstark, Dina MarieTurner, MichelleIzzo, VivianaTutka, PiotrJ. Bidwell, AmyTykkyläinen, MarkkuJaacks, LindsayTytherleigh, MichelleJackson, SaraTytła, MalwinaJackson, NickiTzanis, ChrisJacobs, DavidTziomalos, KonstantinosJacobs, David E.Uhl, EberhardJacobsen, RamuneUlibarri, GerardJacobson, SandraUpshur, Carole C.JafariNasabian, PegahUrban, AlešJager-Hyman, ShariUrrestarazu, ElenaJagnoor, JagnoorUrschler, DavidJahns, LisaUrzi Brancati, CesiraJaine, RichardUsai, AlessiaJaki, BirgitUusi-Rasi, KirstiJakovljevic, MihajloUzu, GaëlleJalava, KatriVaccarini, MassimoJames, AndrewVaccarini, KatiusciaJames, Shirley A.Vacchina, VéroniqueJames-Todd, TamarraVafeiadi, MarinaJanczarek, MonicaVaiman, DanielJandl, RobertVakulin, AndrewJang, SiwonValanne, SusannaJankowska, MartaValentukeviciene, MarinaJanssen, SabineValeo, CaterinaJaradat, AbdullahValério, ElisabeteJasienska, GrazynaValério Monteiro, Magda SofiaJaśkiewicz, MichałVallejo Ruiz, AsierJatoi, IsmailVallone, Donna M.Javed, Arshad AliVallone, DonnaJay, Sarah M.Valtorta, NicoleJayawardene, Wasantha P.Van, Thinh NguyenJazcilevich, ArónVan Den Berg, MaaykenJego, MaevaVan Den Ende, JefJENA, PRASANTVan Der Ende, P(eter). C.Jenkins, Jill A.Van Der Wel, Kjetil A.Jenkins, Rachelvan Dijk, JitseJennings, Wesley G.Van Dijk, Meine PieterJennings, Mary BethVan Doorn, RobertJennings, Charles R.Van Hees, VincentJensen, RogerVan Hoof, JoostJensen, Marina BergenVan Hoof, Joris J.Jensen, Hanne IreneVan Orden, KimberlyJeong, BongjuVan Rossem, LenieJeronimus, Bertus F.Van Stempvoort, Dale R.Jia, Yunyan AndreaVance, MarinaJia, LiliVance, EricJiang, ShanVandenberg, Ann E.Jiang, Heng (Jason)Vanderslice, RobertJiang, ChengshengVanDerslice, JimJiao, MingliVanscheeuwijck, PatrickJiao, JunfengVardasca, RicardoJiao, TifengVarnes, Julia R.Jicha, Gregory A.Varrica, DanielaJie, CuiVasel, Jean-lucJim, LukVasileiou, KonstantinaJiménez, LucíaVaughn, AllisonJiménez, PaulVaz Moreira, IvoneJiménez Rodríguez, M. LourdesVaz-Moreira, IvoneJiménez-Ballesta, RaimundoVázquez Duhalt, RafaelJimenez-Cisneros, BlancaVedøy, Tord FinneJin, YuchangVeeranki, S. PhaniJin, QinhanVega, ClaudiaJin, JianjunVelasco Garrido, Marcialjin, yuanVelasco Ortega, EugenioJin, AndrewVenables, DanJin, LiVenette, Steven J.Jindal, CharulataVenn, BernardJiru, MintesinotVenter, CarinaJo, Young SukVenturelli, MassimoJohnson, NicholasVenturini, ElisaJohnson, AmyVerani, MarcoJohnson, Evan C.Verd, SergioJohnson, NatalieVerdam, MathildeJohnson, Amy K.Verdeguer, FranciscoJohnson, DaynaVergara, Lizandra Garcia LupiJohnston, JillVerhoeven, AdrieJones, AntwanVerhoughstraete, MarcJones, Rena R.Vermeir, GerritJones, BenjaminVerriele, MarieJones, AndrewVerrottï, AlbertoJones, PeterVesper, StephenJones, Rodney P.Vetter, Sylvia HelgaJones, CarwynViazzi, FrancescaJones, MartinVidal, TâniaJonk, YvonneViegas, CarlaJorens, Philippe G.Vieira, JoãoJørgensen, RikkeVijayan, MuraliJoseph, Djenaba A.Vik, TennleyJoshi, PeterVilaro, Melissa J.Joubert, MichaelVillalobos, BiancaJu, XiangqunVillalonga-Olives, EsterJuárez, Isaac Leobardo SánchezVimercati, LuigiJulian, TimVincent, GraceJun, SunghaeViolanti, John M.Jung, KyujinVirtanen, Jorma I.Jung, ChuleuiVisentin, DenisJungbluth, NielsVita, RobertoJurasic, MarianneVitali, MatteoJurewicz, JoannaVitas, Ana IsabelJurikova, TundeVitztum, ColeyJurzik, LarsVivat, BellaJuster, Robert-PaulVivili, PaulaJuszczak, LesławVo, Minh D.Juyoung, LeeVoaklander, Donald C.Kabir, RussellVogt, BrunoKable, JulieVolavka, JanKaeboe, RonnyVolken, ThomasKagan, Calvin M.Volland, GerhardKagawa, TakahideVolpe, RichardKahle, Erin M.Von Lewinski, DirkKahler, David M.Vosvick, Mark A.Kahler, DavidVoudris, VassilisKahlert, RahelVoulvoulis, NickKaido, ToshimiVozza, IoleKaiser, KathrynVuckovic, MilenaKaiser, TillVuillermoz, CecileKakinuma, KatsuyoshiVuorio, AlpoKakiuchi, EmikoVuyst, StijnKaklamanou, DaphneWade, Charles E.Kaleta, DorotaWagenfeld, Amy E.Kalinich, JohnWaibel, ChristianKällén, BengtWaite, SueKalwasińska, AgnieszkaWalabyeki, JulieKambezidis, HarryWalcek, ChrisKamenov, GeorgeWałdykowski, PiotrKamenov, KaloyanWalker, D. CatherineKamigaki, TaroWalker, EllaKamisalic, AidaWalker, Ana PaulaKamiyama, RyutaroWallace, MaeveKamolz, Lars P.Waller, LanceKamouchi, MasahiroWalser, Sandra M.Kan, JinjunWalsh, ShanaKandala, Ngianga-BakwinWalters, VickyKandt, JensWalton, AnnMarie L.Kane, EleanorWalton, AnnMarieKang, Choong-MinWalz, YvonneKang, BumjoonWan, ShupingKang, Sang JunWan, ZhanmingKang, SanghoonWan Mohd Yunus, Wan Mohd AzamKanning, MartinaWanchai, AusaneeKano, MegumiWang, Lian-ShengKansal, MonikaWang, DaowenKantenbacher, JosephWang, YangKanyong, ProsperWang, Zheng-XinKao, Nien-HsinWang, YulingKapellas, KostasWang, Ming-HuaKaplan, BonnieWang, JianyingKaplan, Cameron M.Wang, ChenghaoKappell, AnthonyWang, DiKapuśniak, JanuszWang, JianxuKarabetsos, EfthymiosWang, RuoniuKarachaliou, MariannaWang, DongKarakitsios, SpyrosWang, JinfengKarambelas, AlexandraWang, ZhengxinKaranis, PanagiotisWang, ShigongKarim, Salim AbdoolWang, Grace YKarimli, LeylaWang, JunboKärkkäinen, SirpaWang, MoKarlin, BradleyWang, FengKarpyn, AllisonWang, YinheKarriker-Jaffe, KatherineWang, AolinKasai, NaoyaWang, HaitianKashima, SaoriWang, Hsiao-hsuanKatarina Lilith Johansson, HannaWang, PingKatsoyiannis, AthanasiosWang, WeiKaucic, MassimilianoWang, ShengruiKauffman, Anne Kathryn MarieWang, Li-YuKauko, LeiviskäWang, XiangKaushal, NavinWang, JiashengKavé, GititWang, ZhenshengKawada, ManabuWang, QunKawai, Vivian K.Wang, JiejingKeddell, EmilyWang, LitaoKeegan, ThomasWaples, JamesKeida, ElixabethWarber, SaraKeller, Joshua P.Ward, Kenneth D.Keller, Peter M.Ward, Peter M.Keller, Anita C.Ward, EmmaKellesarian, SergioWark, StuartKelley, DannielleWarmoth, KrystalKelley, RachaelWatanabe, ToruKelly, GabrielleWatanabe, TsunemiKelly, MartinaWaters, Catherine M.Kelly, InasWaters, JaneKelly, Cheryl M.Waters, MatthewKelly, Kevin M.Watkins, KariKemper, Han C. G.Wątróbski, JarosławKempson, Ivan M.Watt, PeterKenkel, DonaldWeaver, ScottKennair, Leif Edward OttesenWeaver, LouiseKennedy, JonathanWeaver, MatthewKennel, JulieWebb, TomKenney, ChristineWebb, Cameron E.Kent, JenniferWebb, ElizabethKepka, Deanna LeeWeber, Ellerie S.Keraminiyage, KaushalWebster, Noah J.Kermanshah, AmirhassanWecht, JillKershaw, Kiarri N.Wege, NataliaKersten, Ellen E.Wei, Chao-YangKersten, Jan FelixWei, BenyongKhalaf, WalaaWei, Da-HuaKhalil, HusseinWei, YeKhalil, ChristianWei, BingganKhamisa, NatashaWeidhaas, JenniferKhan, IzharWeinberg, SethKhan, Jahangir A. M.Weiner, JudithKhanna, SunaliWeiner, Shira SchecterKhanna, SavitaWeis, Megan A.Khayesi, MeleckidzedeckWeismayer, ChristianKhlebnikov, Andrei I.Weiss, SabrinaKhodadoust, Amid P.Weiss, WilliamKhubchandani, JagdishWeitkamp, GerdKhuder, Sadik A.Weker, HalinaKhurram, SyedWelch, DavidKian, RozitaWelden, NatalieKidd, LisaWelgamage Don, AakashKiesel, LisaWelles, SethKietrys, David M.Wells, EllenKilian, ReinholdWells, Brian J.Kim, Harris Hyun-sooWells, NancyKim, JayWells, DeborahKim, Kyung-suWelzel, TysonKim, MinchulWeng, ChingfengKim, BoRinWest, Suzanne L.Kim, DohyeongWesterlund, AnnaKim, JinohWestphal, AndreasKim, Cheol-HoWestrupp, Elizabeth MaryKim, Woo-YangWhite, KimKim, Min JooWhite, NicoleKim, SoJungWhite, Scott M.Kim, Sun-YoungWhite, WendyKim, Woo JinWhitehouse, HilaryKim, Hyoung-RyoulWhiteley, HughKim, Jong InWhittaker, PeterKim, SeonghoWhittemore, KatherineKim, Hyeong SuWickliffe, JeffreyKim, Eun-JinWidenmeyer, MarcKim, Hyun-WooWieczorowska-Tobis, KatarzynaKim, HyunWiedemann, FlorianKim, JinkiWielgomas, BartoszKim, Young-jaeWiernik, Brenton M.Kim, Seong DaeWierzejska, ReginaKimball, Samantha M.Wieser, SimonKimberly, KellyWiesmeier, MartinKinchin, IrinaWiggins, Brenda P.King, TanyaWiggs, MichaelKing, BrynWiklund Axelsson, SarianneKing, Marco-FelipeWilburn, Kathleen M.Kinsky, SuzanneWilde, AnnegretKintziger, KristinaWildman, JohnKirchengast, SylviaWilking, CaraKirilenko, AndreiWilliams, Joah L.Kirkham, M. B.Williams, AdinaKirkpatrick, SharonWilliams, MattKirnan, JeanWilliamson, GrantKirtley, OliviaWilson, Anne M.Kita, ToshikoWilson, Debra RoseKivistö, RauniWilson, PhilipKjellgren, AnetteWilson, Fernando A.Kjellstrom, TordWilson, PeterKlaperski, SandraWilson, Mark J.Klarin, BengtWindham, GayleKlaschka, UrsulaWinford, EboniKlaus, SchäferWinkler, Daniel E.Kleiman, Evan M.Winters, ShannonKlein, IrwinWinters, StephenKlika, RiggsWinwood, PeterKlimes-Dougan, BonnieWirth, MichaelKlimkowicz-Pawlas, AgnieszkaWitorsch, Raphael JayKluczniok, KatharinaWittlich, MarcKnapp, Caprice A.Wohlfarth, RainerKnappett, PeterWójkowska-Mach, JadwigaKnapstad, MaritWollesen, BettinaKneil, KaliWon, Jae WoongKnight, JohnWong, Vincent Kam WaiKnott, RachelWong, Jen D.Knox, OliverWong, Grace Lai-HungKnudsen, Gabriel ArtherWong, Ling TimKobayashi, Natsuko I.Wong, CynthiaKoch, ManfredWoo, Jong MinKociu, ArbenWood, JenKocjančič, TinaWoodfield, LorayneKocot, JoannaWoodrow, ChrisKoda, EugeniuszWoods-Chabane, Gwen C.Koduru, Janardhan ReddyWoods-Townsend, KathrynKoelmans, BartWoodward, Alistair JackKohlitz, JeremyWoolcock, GeoffKohno, KunieWotschack, PhilipKoizumi, HiroyasuWovkulich, KarenKok, GerjoWright, KamauKolar, MilaWright-Berryman, JenniferKolmes, Steven A.Wu, Chih-DaKõlves, KairiWu, YanyanKomljenovic, DraganWu, LiyunKonarski, JanWu, Hung-YiKondo, MichelleWu, JianboKong, In-sooWu, YonghuaKongar, ElifWu, Kevin Chien-ChangKonradsen, HanneWu, WeiningKontogiorgis, ChristosWu, LifengKoo, ChamunWu, QiKoppe, Janna G.Wu​, Alan HbKorajkic, AsjaWuppermann, Amelie C.Koreanschi, AndreeaWynne, OliviaKorhonen, IlkkaWyver, ShirleyKorkeila, JyrkiXi, JunqiangKormas, KonstantinosXia, TingKornhaber, RachelXia, YankaiKorzeniewska, EwaXia, MeimeiKos, BorXia, NingshaoKósa, KarolinaXia, YuchenKosarac, IvanaXiang, YishaKoser, KhalidXiang, JianjunKosmider, LeonXiang, YuKosova, KlaraXiaohui, XuKotula-Balak, MalgorzataXiaoli, AlusKousha, TermehXie, XiaoyaoKovacic, Melinda ButschXie, GraceKovanen, Petri T.Xie, Jin GuiKow, Alfred Wei ChiehXie, ShaohuaKowalska, MałgorzataXing, YangangKowalska-Góralska, MonikaXiong, ZhaohuiKoyama, AsukaXu, XunKoyama, YuXu, YongyongKozisek, FrantisekXu, XiaohuiKraak, VivicaXu, XinKraft, MichaelXu, IrisKrakow, MelindaXu, ZhiweiKral, MichaelXu, YuanKrata, Agnieszka AnnaXuan, Tran DangKraut, AllenXue, JinkaiKrawczyk, MagdalenaYAGER, ERICKrienert, Jessie L.Yamada, TetsujiKrist, LilianYamamoto, NaomichiKristiansen, JesperYang, XiaomeiKristjansson, AlfgeirYang, ZhongpingKroon, JeroenYang, LinshengKroon, Frederieke J.Yang, FengKrüger, FredYang, Kwang IkKrukowski, HenrykYang, XiaKrupinski, Elizabeth A.Yang, YongKrushkal, Julia S.Yang, XinKrysinska, KarolinaYang, MinKuang, Ze-MinYang, FanKucharska-Newton, AnnaYang, HainingKudielka, Brigitte M.Yang, JiachuanKudláček, MichalYannopoulos, StavrosKudłak, BłażejYano, TakashiKudumija Slijepčević, MarijaYao, YuChunKuhfuss, LaureYassi, AnnaleeKuhnlein, Harriet V.Yates, Nathanael J.Kulaga, ZbigniewYe, MingKumanyika, Shiriki K.Yearwood, Edilma L.Kumar, VikasYeckel, Catherine WeikartKumar, RatikaYee, SusanKumar, BinodYeh, Chih-koKumar, KrishanYeh, Chia-TsungKundi, MichaelYeh, Tsu-MingKunert, KarlYeh, Shu-HuiKunst, AntonYen, Yee-wenKuo, Shu-lungYeo, Younsook AnnaKurade, Mayur B.Yershova, KseniyaKurti, Allison N.Yi, Jean C.Kuwahara, KeisukeYiannakoulias, NikoKvaal, KariYiin, Lih-MingKwak, Moon K.Yilmaz, KuzeyKwak, Kyung-HwanYin, JingjingKwan, Mei-PoYing, ZhekangKwok, RichardYokoyama, Yokokwok-Wing, ChauYoo, Do GuenKyne, DeanYoo, Tag KeunKypriotakis, GeorgeYoo, Hong IlKyung, GyouhyungYoon, SukhwanKyzas, George Z.Yoon, JiyoonL. Schmitz, CathryneYoshiki, SyujiLacaze, BernardYoshinaga, JunLadeira, CarinaYoshinaga, JunLåftman, Sara BrolinYoshizumi, TerryLagroye, IsabelleYou, WenLahiri, SriyankaYou, KefeiLai, Julian C. L.You, XiaonaLai, Chia-HsiangYoung, Sean G.Lai, Ka ManYoung, JeanineLaidlaw, MarkYoung, SeanLaird, BrianYousefzadeh, SepidehLake, IainYoussef, NohaLallier, Thomas E.Yperman, JanLam, Tsan YukYu, XiaosongLam, Simon C.Yu, Jin-GangLam, Sean Shao WeiYu, Tsung-HsienLang, IngeborgYu, Chia-YuanLangaas, SindreYu, RongjieLanger, SarkerYu, Jae-HyukLangiano, ElisaYu, RubyLanning, Beth A.Yu, Chia-PinLanou, Amy JoyYuan, QuanLansley, GuyYuan, Chao-QingLantes, OscarYuan, Pao-ChiangLao, ChunhuanYuan, XueLarge, MatthewYuan, HaoranLarkins, NicholasYue, QiLarsen, MarkYue, XiaohangLarson, LincolnYuen, BelindaLarson, BriannaYuen, Hon K.Lassnig, HeimoYurasek, Ali M.László, MakraYurekli, AydaLauerma, HannuZabka, MartinLauridsen, Jørgen TrankjærZaborskis, ApolinarasLauritano, DorinaZabucchi, GiulianoLaverack, GlennZaccheo, PatriziaLavigne, EricZaharia, CarmenLavin, RobertaZalouk-Vergnoux, AuroreLavkulich, Les M.Zambrano, LuisLavoie, Raphael A.Zanini, BarbaraLavoie, KimZarfel, Gernot E.Law, JaneZavadskas, Edmundas KazimierasLaw, EvelynZeiner, MichaelaŁawniczek-Wałczyk, AnnaZeman, Daniel MeierLawrence, ClaireZempila, Melina MariaLawrence, David A.Zeng, GuangmingLawrence, WandaZeng, HuipingLawrenson, RossZeng, DiLaws, Michael BartonZeng, ShihongLawson, Joshua A.Zeni, OlgaLawson, JustinZhang, JianLaxe, SaraZhang, WeiLazarin, AngélicaZhang, JayLazzerini, MarziaZhang, ZengqiangLe, HuongZhang, TaoLe, Cuong NguyenZhang, JingwenLeach, HeatherZhang, ChenLeader, AviZhang, BingLeary, EmilyZhang, YushengLeavy, JustineZhang, QuinLebeck, JanneZhang, YangLebel, AlexandreZhang, PanyueLeboeuf, GaelZhang, ZhiyongLeClair, Mark S.Zhang, JunzengLedda, MarioZhang, DongfengLee, Joe-MingZhang, YingLee, Young JiZhang, DeqiangLee, JonghoZhang, ZhuoLee, EunsuZhang, HaifengLee, Eul-BumZhang, RongjunLee, Paul H.Zhang, AiyongLee, Regina L. T.Zhang, ZhenhuaLee, JosephZhao, XianhuiLee, Nora LZhao, YunLee, Jae EunZhao, YaqianLee, Gyu-CheolZhao, WeifengLee, JoonZhao, QiranLee, Won JinZhao, YichuanLee, Jin-YongZhao, WenbingLee, Joseph G. L.Zheng, XiaoyingLee, Jong SeonZheng, TongzhangLee, PetronaZhong, WeihongLee, JiyoungZhou, LifengLee, Ho-WonZhou, QingxiangLee, Wing-KeeZhou, LuxiLee, Jae SeungZhou, An-NanLee, Do-GilZhou, ChengchaoLee, Kuo-KauZhou, LigangLee, Huei-JaneZhu, YumingLEE, Yiu Yin RaymondZhu, ZhenduoLee, Yung-JaanZhu, HongLee, Kyung-YilZhu, XiaoshanLees, RosemaryZhu, LinLefering, RolfZhu, ShijunLefterov, IliyaZhuang, JieLegault, LaurentZhuang, ZiqingLeggat, Sandra G.Zhurakhovska, LiliaLehning, AmandaZiegler, JaneLeibovici, DidierZielenkiewicz, UrszulaLeidl, ReinerZielinski-Gutierrez, EmilyLeiherer, AndreasZijlema, WilmaLeisner, JørgenZilberman, UriLeisyte, LiudvikaZimeri, Anne MarieLekies, Kristi SZimmerman, EmilyLelard, ThierryZineldin, MosadLemke, StefanieZou, EnminLenart-Boroń, AnnaZouharova, MonikaLeng, Xiaoyan IrisŹróbek-Sokolnik, AnnaLent, Michelle R.Żubrowska-Sudoł, MonikaLeón, Gerardo A.Zucca, PaoloLeón Pérez, Jose MariaZüllig, RichardLeonard, AnneZuniga-Teran, AdrianaLeone, AntonioZUSMAN, EricLeone, Matthew C.Zweifel, PeterLeong, David TaiZwoździak, Anna

